# Sacral Stress Fracture in an Amateur Badminton Player

**DOI:** 10.1155/2017/4268981

**Published:** 2017-10-03

**Authors:** Yusuke Yuasa, Naohisa Miyakoshi, Michio Hongo, Kunio Ebata, Tatsuru Tomioka, Yoichi Shimada

**Affiliations:** ^1^Department of Orthopedic Surgery, Yokote Municipal Hospital, 5-31 Negishi, Yokote 013-8602, Japan; ^2^Department of Orthopedic Surgery, Akita University Graduate School of Medicine, 1-1-1 Hondo, Akita 010-8543, Japan

## Abstract

Sacral stress fractures are rare among athletes but have been reported most frequently in long distance runners. We report herein the first case of a sacral stress fracture in an amateur badminton player. A 16-year-old, left-handed adolescent girl, who had just started to play badminton 3 months previously, complained of acute left buttock pain when she received a shuttlecock. Magnetic resonance imaging revealed a linear lesion of the left sacrum with low signal intensity on T1- and high signal intensity on T2-weighted images, which was consistent with a stress fracture. Conservative treatment with rest relieved her symptoms. Her fracture was considered to have occurred due to repetition of an exercise that caused excessive vertical power.

## 1. Introduction

Sacral stress fractures are rare among athletes. Previous reports have shown that the fractures most frequently occur in long distance runners [1, 2]. Stress fractures of the sacrum have also been sporadically reported in a hockey player, tennis player, soccer player, and volleyball player [3–6]. However, to the best of our knowledge, the fracture has not been reported in a badminton player. We herein describe a case of sacral stress fracture in an amateur badminton player.

## 2. Case Presentation

A 16-year-old, left-handed adolescent girl presented with left buttock pain that had persisted for 1 month. She had just started to play badminton 4 months prior.* She had been playing for 2 or 3 hours a day. She had been experiencing gradually increasing pain on her left buttock without noticing any cause. The pain was generated every time when she received a shuttlecock close to the net while stepping forward with her left knee bent*.

On physical examination, her buttock pain was generated when she moved her hip joint and when she lay down on her back. Patrick test was positive on the left side. She had tenderness of the left sacroiliac joint area but not of the anterior hip. Straight leg raise test and Newton test were negative bilaterally. Fingertip-to-floor distance when she bent forward was 0 cm.

**Figure 1:**
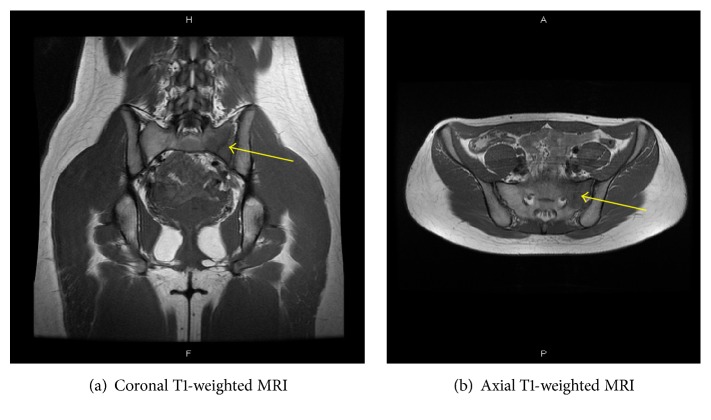
T1-weighted MRI scan shows a linear area of decreased signal intensity of the left sacrum with surrounding a T1 hypointense area (arrow).

**Figure 2:**
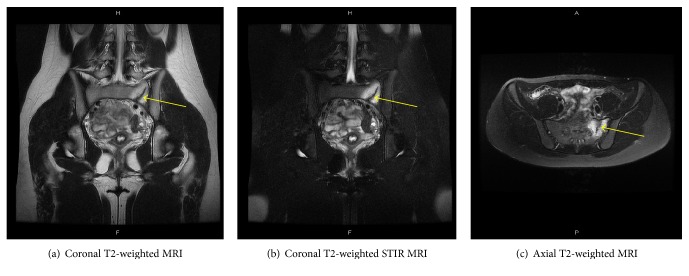
MRI T2 and T2 short tau inversion recovery (STIR) scan shows a linear area of decreased signal intensity of left sacrum with surrounding a T2 hyperintense area (arrow).

On laboratory testing, serum calcium and phosphorus and renal and liver functions were within normal limits. A plain radiograph of the pelvis showed a mild degree of acetabular dysplasia on both sides. Magnetic resonance imaging (MRI) revealed a linear lesion of the left sacrum with low signal intensity on T1- (Figure 1) and high signal intensity on T2-weighted images (Figure 2).* No signal change of the intraarticular lesion including labrum tears of the hip joint was found*. Her bone mineral density compared with the young adult mean was 100% for the L2–L4 spine and 121% for the right proximal femur.

Based on these clinical and radiological findings, we diagnosed the cause of the pain to be a stress fracture of the sacrum. The patient was instructed to stop sports participation until she was free of pain. Three months after discontinuing sports activities her pain disappeared, and she returned to playing badminton.

## 3. Discussion

Badminton injuries most frequently involve the lower limbs, especially the knees, and almost all overuse injuries occur in the lower limbs [7]. A case of stress fracture of the second metacarpal bone has been reported in a badminton player [8]. Sacral stress fractures have been reported mostly in long distance runners and also in hockey, soccer, tennis, and volleyball players [1–6].* There has been no reported case of a badminton-related sacral stress fracture yet*. This is the first reported case of a badminton-related sacral stress fracture.

There are sacroiliac joint pain syndromes and hip joint diseases as possible causes of buttock pain. In this case, a hip joint disease was excluded because she did not have tenderness of the anterior hip, and no signal change of the intraarticular lesion was found in MRI. The pain originated from the sacroiliac joint pain syndromes was usually diagnosed with several clinical symptoms, but not with MRI alone. This case was diagnosed as a sacral stress fracture because there was obvious fracture line of the sacrum on MRI.

**Figure 3:**
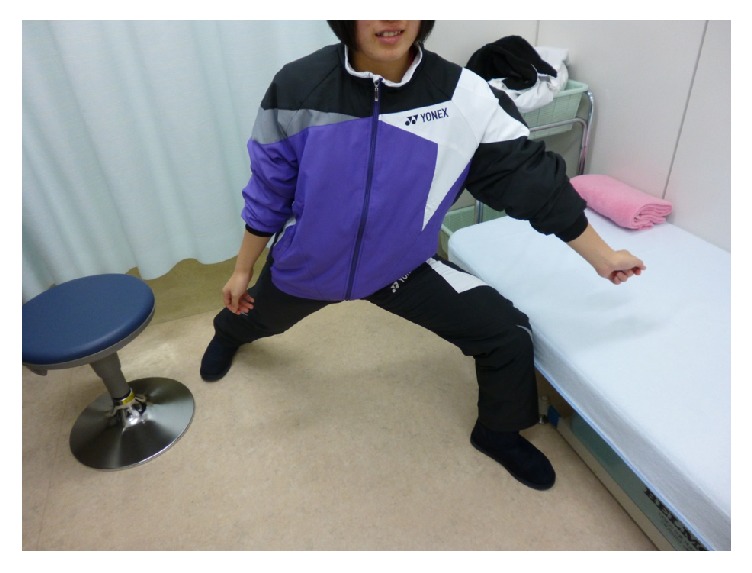
This picture is underhand stroke posture.

In badminton, there are several ways to hit the shuttlecock, that is, overhead and underhand strokes. The overhead stroke is used to hit a shuttlecock falling from higher than the player's height. The player can transmit power to a shuttlecock during the hit by shifting one's weight from the dominant foot and moving forward to the nondominant side. On the other hand, the underhand stroke is used to pick up a returned shuttlecock slowly dropping near the net at the front of the court (Figure 3). The player returns the shuttlecock while standing on one's dominant foot with the trunk erect. This repetitive motion might have induced mechanical stress on our patient's dominant side. During such movement, vertical power is applied to the sacrum on the dominant side, and the sacrum is exposed to repetitive stress, which in this case may have induced the stress fracture. Besides, the fracture occurred unilaterally. In a biomechanical analysis by Linstrom and others [9] it was suggested that the asymmetric stress transmission to the sacrum through iliac bone might cause unilateral vertical sacral fractures.

We considered several possible potential factors as causes of the stress fracture, such as an abnormal menstrual history, dietary deficiencies, and low bone mineral density [10]. In the current case, no underlying diseases affecting bone metabolism were evident. Kahanov and others reported a case of a sacral stress fracture in a distance runner who had tightness of the hamstrings when the intensity of training was increased [1]. Tzoanos and others reported that decreased shock absorption on an artificial surface in a middle-aged soccer player might have influenced the development of sacral fracture [4]. Silva and others described that repetitive movements in baseline strokes and frequent changes in direction were considered as the causes of sacral stress fractures in a tennis player [5]. The fracture in the present case was considered to have occurred due to repetition of an exercise that caused excessive vertical power. Therefore, proper stretching before sports activity and preventing excessive repetition of motion to the front of the court may be effective in preventing stress fractures in badminton players.

## References

[1] Kahanov L., Eberman L., Alvey T., True J., Yeargin B. (2011). Sacral stress fracture in a distance runner. *Journal of the American Osteopathic Association*.

[2] Rodrigues L. M. R., Ueno F. H., Valesin Filho E. S., Fujiki E. N., Milani C. (2009). Sacral stress fracture in a runner: a case report. *Clinics*.

[3] Southam J. D., Silvis M. L., Black K. P. (2010). Sacral stress fracture in a professional hockey player: a case report.. *Orthopedics*.

[4] Tzoanos G., Tsavalas N., Manidakis N., Karantanas A. (2013). Sacral fatigue fracture in an amateur soccer player. *Case Reports in Medicine*.

[5] Silva R. T., De Bortoli A., Laurino C. F. S., Abdalla R. J., Cohen M. (2006). Sacral stress fracture: an unusual cause of low back pain in an amateur tennis player. *British Journal of Sports Medicine*.

[6] Shah M. K., Stewart G. W. (2002). Sacral stress fractures: an unusual cause of low back pain in an athlete. *Spine*.

[7] Goh S. L., Mokhtar A. H., Mohamad Ali M. R. (2013). Badminton injuries in youth competitive players. *Journal of Sports Medicine and Physical Fitness*.

[8] Fukuda K., Fujioka H., Fujita I. (2008). Stress fracture of the second metacarpal bone in a badminton player. *Kobe Journal of Medical Sciences*.

[9] Linstrom N. J., Heiserman J. E., Kortman K. E. (2009). Anatomical and biomechanical analyses of the unique and consistent locations of sacral insufficiency fractures. *Spine*.

[10] Johnson A. W., Weiss C. B., Stento K., Wheeler D. L. (2001). Stress fractures of the sacrum. An atypical cause of low back pain in the female athlete. *The American Journal of Sports Medicine*.

